# Differences in systemic and mucosal SARS-CoV-2 antibody prevalence in a prospective cohort of Dutch children

**DOI:** 10.3389/fimmu.2022.976382

**Published:** 2022-09-09

**Authors:** Maya W. Keuning, Marloes Grobben, Merijn W. Bijlsma, Beau Anker, Eveline P. Berman-de Jong, Sophie Cohen, Mariet Felderhof, Anne-Elise de Groen, Femke de Groof, Maarten Rijpert, Hetty W. M. van Eijk, Khadija Tejjani, Jacqueline van Rijswijk, Maurice Steenhuis, Theo Rispens, Frans B. Plötz, Marit J. van Gils, Dasja Pajkrt

**Affiliations:** ^1^ Department of Pediatric Infectious Diseases, Rheumatology & Immunology, Amsterdam University Medical Centers (UMC), location University of Amsterdam, Amsterdam, Netherlands; ^2^ Department of Medical Microbiology and Infection Prevention, Laboratory of Experimental Virology, Amsterdam University Medical Centers (UMC) location University of Amsterdam, Amsterdam, Netherlands; ^3^ Infectious diseases, Amsterdam Institute for Infection and Immunity, Amsterdam, Netherlands; ^4^ Department of Pediatrics, Emma Children’s Hospital Amsterdam University Medical Centers (UMC), University of Amsterdam, Amsterdam, Netherlands; ^5^ Department of Pediatrics, Flevoziekenhuis, Almere, Netherlands; ^6^ Department of Pediatrics, Noordwest Ziekenhuisgroep, Alkmaar, Netherlands; ^7^ Department of Pediatrics, Zaans Medical Center, Zaandam, Netherlands; ^8^ Department of Immunopathology, Sanquin Research, Amsterdam, Netherlands; ^9^ Landsteiner Laboratory, Amsterdam University Medical Centers (UMC), University of Amsterdam, Amsterdam, Netherlands; ^10^ Department of Pediatrics, Tergooi Medical Center, Blaricum, Netherlands

**Keywords:** SARS-CoV-2, mucosal antibody response, mucosal IgG, antibody prevalence, children, saliva antibodies

## Abstract

**Background:**

As SARS-CoV-2 will likely continue to circulate, low-impact methods become more relevant to monitor antibody-mediated immunity. Saliva sampling could provide a non-invasive method with reduced impact on children. Studies reporting on the differences between systemic and mucosal humoral immunity to SARS-CoV-2 are inconsistent in adults and scarce in children. These differences may be further unraveled by exploring associations to demographic and clinical variables.

**Methods:**

To evaluate the use of saliva antibody assays, we performed a cross-sectional cohort study by collecting serum and saliva of 223 children attending medical services in the Netherlands (irrespective of SARS-CoV-2 exposure, symptoms or vaccination) from May to October 2021. With a Luminex and a Wantai assay, we measured prevalence of SARS-CoV-2 spike (S), receptor binding domain (RBD) and nucleocapsid-specific IgG and IgA in serum and saliva and explored associations with demographic variables.

**Findings:**

The S-specific IgG prevalence was higher in serum 39% (95% CI 32 – 45%) than in saliva 30% (95% CI 24 – 36%) (P ≤ 0.003). Twenty-seven percent (55/205) of children were S-specific IgG positive in serum and saliva, 12% (25/205) were only positive in serum and 3% (6/205) only in saliva. Vaccinated children showed a higher concordance between serum and saliva than infected children. Odds for saliva S-specific IgG positivity were higher in girls compared to boys (aOR 2.63, P = 0.012). Moreover, immunocompromised children showed lower odds for S- and RBD-specific IgG in both serum and saliva compared to healthy children (aOR 0.23 – 0.25, P ≤ 0.050).

**Conclusions:**

We showed that saliva-based antibody assays can be useful for identifying SARS-CoV-2 humoral immunity in a non-invasive manner, and that IgG prevalence may be affected by sex and immunocompromisation. Differences between infection and vaccination, between sexes and between immunocompromised and healthy children should be further investigated and considered when choosing systemic or mucosal antibody measurement.

## Introduction

Severe acute respiratory syndrome coronavirus 2 (SARS-CoV-2) will likely continue to circulate in the coming years. In the context of ongoing public health measures and vaccination programs, it is crucial to keep monitoring humoral immunity. Children have not been equally represented in immunosurveillance, while they do play a role in the transmission of SARS-CoV-2 (1). Surveillance of immunity in children is important to establish effective public health measures for this group. However, as the urgency of the pandemic decreases, it becomes more relevant to develop non-invasive methods to monitor antibody-mediated immunity to reduce impact on children and improve the willingness to participate. Coronavirus disease 2019 (COVID-19) convalescent patients and vaccinated individuals produce both serum and mucosal antibodies (2, 3). Although serum antibodies are traditionally measured, saliva sampling for mucosal antibody measurements has shown promising first results leading to the first FDA approved saliva based antibody test in June 2021 (4, 5). As saliva sampling is quick and painless this could be a convenient alternative to serum sampling, in particular for children.

SARS-CoV-2 infection in the upper airway induces local innate and adaptive immune responses in the mucosa (6). Evidence is growing that mucosal immunity, particularly through neutralizing antibodies, is important to control SARS-CoV-2 infection (7–9). Mucosal and systemic immunity can function as separate compartments, but each can influence the other as well (10, 11). As a result, both locally produced and systemically derived antibodies can be detected in the mucosa (12). Although IgA is the most abundant isotype in mucosal surfaces, previous studies have supported the assumption that salivary IgG, which is mostly derived from the systemic compartment, is better suitable to detect previous SARS-CoV-2 exposure than salivary IgA (13–15). The growing interest in mucosal immunity also stimulated the development of mucosal vaccines or therapeutic interventions, for which measuring the locally induced mucosal immune response will become even more important (6, 16).

The development of mucosal assays or interventions is hampered by the lack of comprehensive understanding of mucosal immunity and its relation to systemic immunity (17). Moreover, current literature comparing mucosal and systemic humoral immunity is inconsistent. While some studies have shown high prevalence of mucosal antibodies with similar durability to serum in convalescent patients (2, 15), others reported lower proportions of patients with detectable mucosal antibodies as compared to serum (18). In adult patients, quantity and durability of serum antibodies have been associated with sex or comorbidity (19, 20). Evidence on associations of mucosal antibodies to sex, age or comorbidity is lacking or contradictory (7, 14, 21). In addition, associations of humoral responses and demographic variables in children are rarely described. Differences in the induction and durability of systemic and mucosal humoral immunity may be unraveled by exploring these associations.

Prevalence studies among populations with a combination of natural and vaccine-induced immunity against SARS-CoV-2 are of value in evaluating the performance of mucosal antibody assays in the whole population. In our previous cohort during the first wave of the COVID-19 pandemic, we detected a low prevalence of SARS-CoV-2 specific IgG in children with differences in the presence of mucosal and systemic antibodies (22). In the current prospective cross-sectional study, we describe higher prevalence for serum and saliva SARS-CoV-2 antibodies than in our previous cohort, and we further explore heterogeneity between serum and saliva by evaluating associations with demographic and clinical variables. We show that tracking humoral immunity through saliva-based assays could be useful for identifying SARS-CoV-2 naïve populations and vaccine responses.

## Materials and methods

### Study design

For this cross-sectional study we simultaneously sampled blood and saliva of children attending medical care at six secondary and tertiary care hospitals in the North-West region of the Netherlands during May 10^th^ to October 15^th^ 2021. All children aged 0 to 18 years requiring blood testing or intravenous cannulation for any reason were eligible. Eligibility was irrespective of a (suspected) acute or prior SARS-CoV-2 infection.

### Study definitions

History of previous positive PCR- or rapid antigen test for SARS-CoV-2 infection and COVID-19 vaccination status were collected to distinguish population subgroups. During the inclusion period of this study, the Health Council of the Netherlands announced their recommendation for COVID-19 vaccination in children ≥ 12 years of age with comorbidity. For analysis between population subgroups, inclusion 14 days after infection or vaccination was considered sufficient for a detectable antibody response (9, 23). Children infected or vaccinated within 14 days prior to inclusion or with both a previous infection and vaccination were excluded from analysis within the population subgroups. Severity of SARS-CoV-2 infection was classified into five categories (asymptomatic, mild, moderate, severe and critically severe) of COVID-19 as published by Dong et al. or Multi-Inflammatory Syndrome in Children (MIS-C) based on patient-reported clinical features (24). Children with immunodeficiency, autoimmune disease, hematological malignancy and/or use of immunomodulating drugs were defined as having an ‘immunocompromised state’. Immunomodulating drugs included: azathioprine, methotrexate, monoclonal antibodies, immunoglobulins and corticosteroids. We defined children with an ‘underlying illness’ as children with obesity, respiratory, cardiovascular, endocrine, metabolic, hematologic, or kidney diseases, solid malignancies, or psychomotor retardation. Previously healthy children were categorized as having ‘no relevant medical history’. Obesity was defined for children aged 2 to 5 years as weight-for-length z-score + 3 standard deviations (SD) and for children aged 5 to 18 years as BMI-for-age z-score + 2 SD (25).

### Sample collection

Methods of serum and saliva sampling and analyses were as previously described (22). In short, during venipuncture a blood sample of 1 to 5 ml was collected, centrifuged and serum was stored at -20° C. Saliva was obtained by passive drooling directly into a sterile container or *via* a buccal swab (ORACOL Saliva Collection Device, Product Code S10, Malvern Medical Developments Ltd) from which the saliva was extracted into a sterile tube by centrifugation. The resulting samples were centrifuged at 1000 rpm for 10 minutes and stored at -80° C.

### Luminex assays

A Luminex assay was developed to determine SARS-CoV-2 specific antibodies in serum and saliva as described previously (22). SARS-CoV-2 spike (S), receptor binding domain of the spike (RBD) and nucleocapsid (N) antigens were covalently coupled to Luminex MagPlex beads. Fifteen of each SARS-CoV-2 antigen coupled bead per µl was incubated with 1:10,000 diluted serum or 1:10 diluted saliva at a 1:1 ratio and incubated overnight at 4°C. The next day, washing was followed by a two-hour incubation with goat anti-human IgG-PE or goat anti-human IgA-PE (Southern Biotech). After washing, detection was performed on a MAGPIX instrument (Luminex). Read-out was expressed as the median fluorescence intensity (MFI) of at least 50 beads per antigen. Positive and negative control beads were included in every well. To control for variation between plates, positive and negative control sera or saliva samples were included on every plate as well as a titration of serum or saliva of a known SARS-CoV-2 infected patient. The cut-offs for IgG antibody prevalence in each assay were established previously (22) and were further supported by testing serum of pre-pandemic (n = 113) or PCR-confirmed SARS-CoV-2 infected adults (n = 282) and testing pre-pandemic saliva samples of children (n = 50) or SARS-CoV-2 infected adults (n = 70) resulting in the sensitivity and specificity values presented in **Supplementary Table 1**. For IgA antibodies in saliva, the previously determined cut-offs were unsuitable due to low sensitivity. Instead, cut-offs were selected after ROC analysis, using pre-pandemic saliva samples of children (n = 50) and SARS-CoV-2 infected adults (n = 70), as the highest sensitivity achievable with a specificity of at least 80% (**Supplementary Figure 1**).

Additionally, we measured serum prevalence with the FDA approved Wantai SARS-CoV-2 RBD total antibody enzyme-linked immunosorbent assay to assess comparability between assays and between the prevalence in this study and other studies. Assays were performed following the manufacturer’s instructions, providing a sensitivity of 97% (95% confidence interval [CI], 83 - 99%) and specificity of 98% (95% CI, 91 - 99%) (26).

### Statistics

At the start of inclusion of this cohort, an S-specific total Ig seroprevalence of 32% was reported among Dutch adult blood donors during national surveys (27), while seroprevalence among children was unknown. A minimum sample size of 214 participants was calculated to measure an expected S-specific IgG seroprevalence of 15% in our cohort with a 95% CI between 10% and 20%.

All statistical analyses were performed in IBM SPSS Statistics version 26 predictive analytics software. Prevalence estimates were calculated as the proportion of participants of the total cohort with SARS-CoV-2 specific IgG above the cut-off for positivity. 95% CI was calculated with the Clopper-Pearson method (28). T-tests and Mann-Whitney U tests were used to compare means and mean ranks across subgroups, and paired t-tests for comparisons of paired groups. Differences in proportion were tested with the Chi-square or Fisher’s exact test and with McNemar test for paired proportions. Pearson correlation coefficients were determined for time since infection and antibody levels in serum and saliva and Spearman’s rank-order correlations for correlations between serum and saliva antibodies. To study the associations between demographic and clinical variables and log transformed serum and saliva SARS-CoV-2 specific IgG, linear regression was performed only for children with antibody levels above the detection limit. Uni- and multivariable logistic regression were performed with serum and saliva SARS-CoV-2 specific IgG antibody prevalence and demographic or clinical variables. Cases with missing data for variables in the regression analysis were excluded. We identified several pre-defined demographic (age, sex) and clinical (comorbidity, COVID-19 vaccination and history of PCR or rapid antigen test positive infection) variables with clinical importance and/or a P < 0.250 in univariable regression analysis. These were included in the models after checking for multicollinearity using Variance Inflation Factors. Data are described as unadjusted and adjusted odds ratios (OR) with 95% CI. In antibody analyses of COVID-19 vaccinated children, only S- and RBD-specific IgG are reported with exclusion of N-specific IgG since N-specific IgG is not induced after vaccination.

### Study approval

The study protocol was approved by the ethics committee of the Amsterdam University Medical Centers (NL73556.018.20). We obtained written informed consent from parents/guardians and/or from children above the legal age of consent.

### Role of the funding source

The funding source stated in the acknowledgement section have financially supported the use of the assays in this study. The funding source did not have a role in the analysis or interpretation of the data, nor in developing and submitting the manuscript.

## Results

### Study participants

Characteristics of the 223 included children are shown in **Table 1**. Paired serum and saliva samples were available for 205 participants. Median age was 13 years with a range of 0 to 18 years and 50% of all children were female. Most children had an immunocompromised state (58%) while 27% reported another underlying illness and 15% reported no relevant medical history.

**Table 1:** Study population characteristics.

	Total cohort	Unknown exposure group	Infected group	Vaccinated group
**Total N** ** Serum samples** ** Saliva samples**	223 212 216	155 147 149	27 26 26	26 24 26
**Sex** ** Female** ** Male**	112 (50%) 111 (50%)	69 (45%) 86 (55%)	17 (63%) 10 (37%)	17 (65%) 9 (35%)
**Age (years)** ** < 1** ** 1-4** ** 5-9** ** 10-14** ** 15-17**	9 (4%) 13 (6%) 39 (18%) 77 (35%) 85 (38%)	8 (5%) 11 (7%) 34 (22%) 62 (40%) 40 (26%)	0 2 (7%) 5 (19%) 5 (19%) 15 (56%)	0 0 0 8 (31%) 18 (69%)
**Inclusion month** ** May** ** June** ** July** ** August** ** September** ** October**	34 (15%) 86 (39%) 17 (8%) 34 (15%) 40 (18%) 12 (5%)	28 (18%) 65 (42%) 12 (8%) 19 (13%) 25 (16%) 6 (4%)	5 (19%) 15 (56%) 1 (4%) 2 (8%) 3 (11%) 1 (4%)	0 2 (8%) 1 (4%) 11 (42%) 7 (27%) 5 (19%)
**Immunocompromised state** - **Immunodeficiency** - **Autoimmune disease** - **Hematological malignancies** - **Use of immunomodulating drugs** **Underlying illness** - **Obesity** - **Respiratory** - **Cardiovascular** - **Neurological** - **Hematologic** - **Kidney disease** - **Endocrine/metabolic** - **Other disease** **No relevant medical history**	130 (58%) 4 (2%) 125 (56%) 2 (1%) 131 (59%) 59 (27%) 21 (9%) 5 (2%) 6 (3%) 5 (2%) 10 (5%) 11 (5%) 11 (5%) 6 (3%) 34 (15%)	84 (54%) 4 (3%) 79 (51%) 2 (1%) 84 (54%) 42 (27%) 12 (9%) 3 (2%) 2 (1%) 5 (3%) 10 (7%) 6 (4%) 8 (5%) 4 (3%) 29 (19%*)*	16 (59%) 0 16 (59%) 0 16 (59%) 8 (30%) 2 (7%) 2 (7%) 1 (4%) 0 0 2 (7%) 1 (4%) 1 (4%) 3 (11%)	18 (70%) 0 18 (69%) 0 19 (73%) **7 (27%)** 5 (19%) 0 2 (8%) 0 0 1 (4%) 2 (8%) 1 (4%) 1 (4%)
**COVID-19 vaccination** - **Not vaccinated** - **Received 1 dose** - **Received 2 doses** - **Data missing**	182 (82%) 19 (9%) 19 (9%) 3 (1%)	154 (100%) 0 0 N/A	26 (100%) 0 0 N/A	0 8 (31%) 18 (69%) N/A
**Type of hospital visit** - **Day-care** - **Outpatient** - **ER visit** - **Inpatient**	183 (82%) 22 (10%) 4 (2%) 14 (6%)	122 (79%) 18 (11%) 3 (2%) 12 (8%)	23 (85%) 2 (7%) 1 (4%) 1 (4%)	24 (92%) 2 (8%) 0 0
**Type of hospital** - **University hospital** - **Non university hospital**	182 (82%) 41 (18%)	126 (81%) 29 (19%)	22 (81%) 5 (19%)	23 (88%) 3 (12%)
**History of PCR proven SARS-CoV-2 infection** - **Yes** - **No** - **Clinical symptoms but tested negative or not tested** - **Data missing** **Hospital admission** - **Yes** - **No** **ICU admission** - **Yes** - **No** **SARS-CoV-2 infection severity** - **Asymptomatic** - **Mild** - **Moderate** - **Severe** - **Critically severe** - **Data missing**	34 (15%) 163 (73%) 11 (5%) 15 (7%) 1 (0.4%) 216 (97%) 1 (0.4%) 217 (97%) Total N= 34 6 19 1 0 1 7	34 (15%0 132 (85%) 9 (6%) 0 N/AN/AN/A	34 (15%27 (100%) 0 0 0 1 (4%) 20 (74%) 1 (4%) 26 (96%) Total N= 27 5 14 1 0 1 5	34 (15%0 26 (100%) 0 0 N/AN/AN/A
**COVID-19 in household** - **Yes** - **No**	40 (18%) 181 (81%)	17 (12%) 137 (88%)	16 (59%) 11 (41%)	3 (12%) 23 (85%)

Characteristics are described for the total cohort (n = 223) and for each study population subgroup. COVID-19, coronavirus disease 2019; ER, emergency room; ICU, intensive care unit; N/A, not applicable; N., number; PCR, polymerase chain reaction assay; SARS-CoV-2, severe acute respiratory syndrome coronavirus 2.

Data on history of SARS-CoV-2 infection was available for 93% (208/223) of the total cohort. A positive PCR- or rapid antigen test for SARS-CoV-2 at least 14 days prior to inclusion was reported in 16% (34/208) of children. Of the SARS-CoV-2 infected children, 6/34 reported an asymptomatic infection, 19/34 a mild infection, 1/34 a moderate infection and 1/34 was a MIS-C patient. SARS-CoV-2 related hospital admission was reported for the MIS-C patient only, who did not require respiratory support but did require ICU admission for circulatory support. Another 5% (11/208) reported a suspicion of COVID-19 but had not been tested or was tested negative. One child received one vaccination prior to infection. Vaccination status was available for 99% (221/223). Of these 221 patients, 18% was vaccinated (6% received one dose at least 14 days prior to inclusion and 9% had received two doses). The median time since previously reported SARS-CoV-2 infection was six months (176 days) and median time since last COVID-19 vaccination was one month (30 days).

### Prevalence and levels of S, RBD and N-specific antibodies

**Figure 1:**
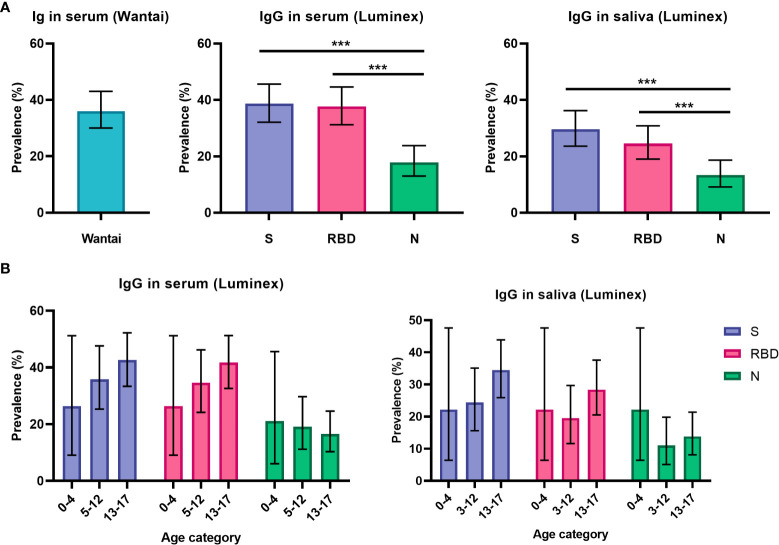
Prevalence of SARS-CoV-2 specific antibodies in serum and saliva. **(A)** Prevalence estimates of RBD-specific antibodies in serum using the Wantai assay (n = 209) and of S-, RBD- and N-specific antibodies using the Luminex assay in serum (n = 212) and saliva (n = 216) for all children (total cohort n = 223). **(B)** Prevalence of S-, RBD- and N-specific antibodies in all children, shown separately for pre-school children (0-4 years old, n = 19 for serum, n = 18 for saliva), primary school children (5-12 years old, n = 78 for serum, n = 82 for saliva) and secondary school children (13-17 years old, n = 115 for serum, n = 116 for saliva). Prevalence estimates are the calculated proportion with a value above the determined cut-off. Estimates are shown with 95% confidence intervals. McNemar test was used for differences between paired proportions. S, spike; RBD, receptor binding domain of the spike; N, nucleocapsid; *** = P ≤ 0.001.

With the FDA approved Wantai assay, seroprevalence was 36% (75/209, 95% CI 29 – 43%, **Figure 1**) for all participants. The Wantai RBD total antibody assay and the Luminex assay for serum RBD-specific IgG were in high agreement (96%); for further comparison of antibody levels and prevalence only Luminex serum assay results are reported. Levels of IgG and IgA antibodies in the Luminex assay are reported in **Supplementary Figure 2**. We observed heterogeneity in the S- RBD- and N-specific IgG prevalence: seroprevalence was higher for S-specific IgG; 39% (82/212, 95% CI 32 – 45%) and RBD-specific IgG; 38% (80/212, 95% CI 31 – 44%) compared to N-specific IgG; 18% (38/212, 95% CI 13 – 24%) in the Luminex assay (**Figure 1**) (P < 0.001). In saliva, we similarly observed heterogeneity, as the antibody prevalence was 30% (64/216, 95% CI 24 – 36%), 25% (53/216, 95% CI 19 – 31%) and 13% (29/216, 95% CI 9 – 19%) for S-, RBD- and N-specific IgG, respectively. This was also significantly lower for N-specific IgG compared to S- and RBD-specific IgG (P < 0.001, **Figure 1**). Since N-specific IgG is only elicited by infection and not by vaccination, we additionally evaluated this heterogeneity in only unvaccinated children. The difference between N-specific IgG compared to S- and RBD-specific IgG was similarly significant in serum (P < 0.008), but not in saliva (P > 0.050, **Supplementary Figure 3**). In line with our previous study findings, sensitivity and specificity of saliva IgA was lower than saliva IgG to detect positive and negative control samples (**Supplementary Figure 1**). Since the highest sensitivity achievable with a specificity of at least 80% was 45 – 75% for SARS-CoV-2 specific IgA in saliva, we did not calculate prevalence for IgA antibodies.

**Figure 2:**
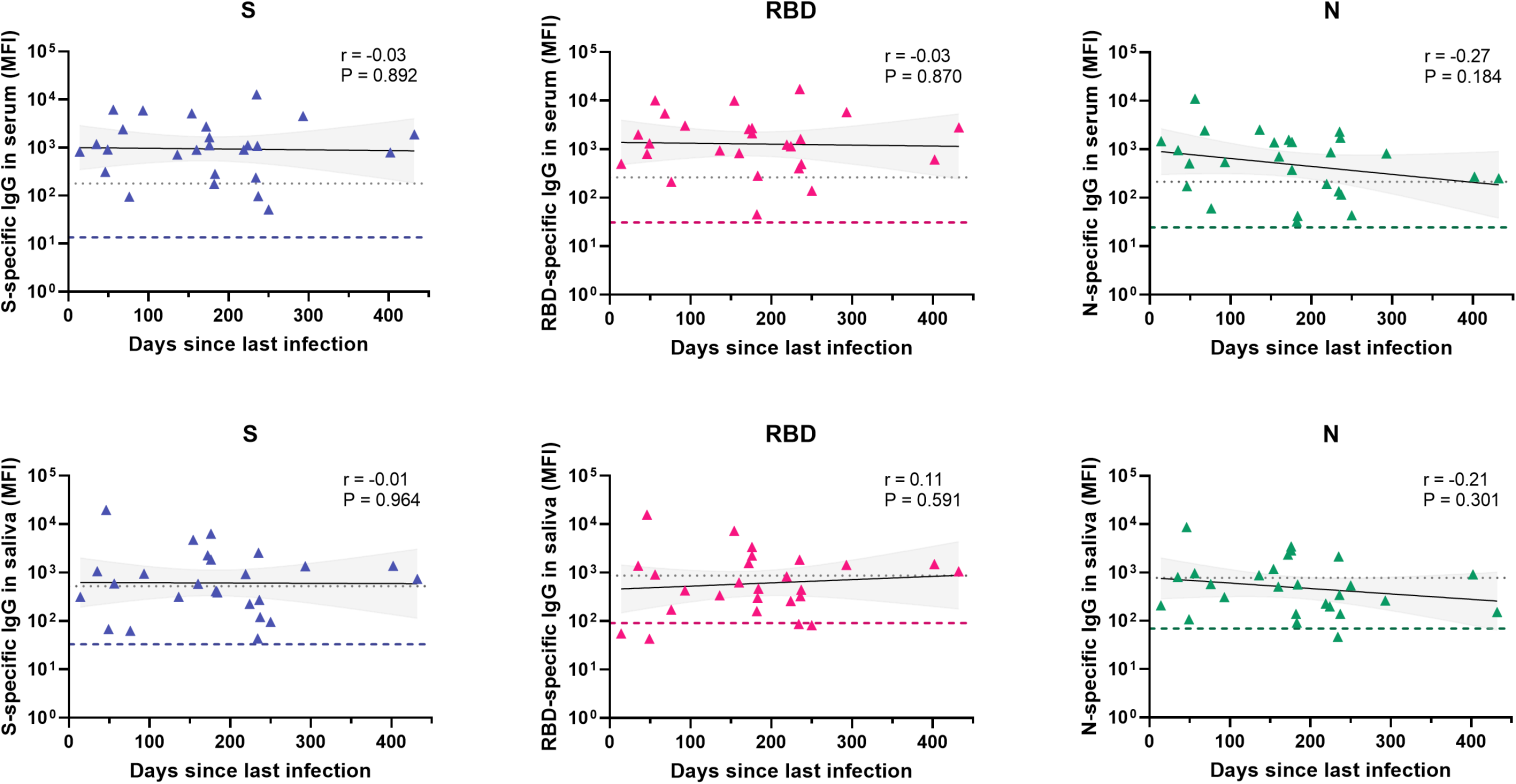
Antibody levels over time for different antigens in serum and saliva after infection. Levels of S-, RBD- and N-specific antibodies in serum (n = 26) and saliva (n = 26) of unvaccinated children with confirmed SARS-CoV-2 infection and known time of infection (total n = 27). Within each graph, each data point is a different individual. The black line represents a linear regression and the grey area the 95% confidence intervals. Pearson correlations were performed and the coefficient (r) and the P-value are shown for each graph. The grey dotted line indicates the assay cut-off and the colored dashed line represents the median MFI of all children in the unknown exposure group as a reference. S, spike; RBD, receptor binding domain of the spike; N, nucleocapsid; MFI, median fluorescence intensity.

Antibody prevalence increased only for S- and RBD-specific IgG with age in the total cohort, although differences were not significant and absolute numbers for pre-school children were low (**Figure 1B**). To further investigate the observed difference between S-, RBD- and N-specific antibody prevalence, we evaluated antibody levels over time since infection for children with a SARS-CoV-2 infection at least 14 days prior to inclusion (n = 27, **Figure 2**). There was a decreasing trend for N-specific antibodies up to 432 days after infection, although there were no significant correlations between time and any antigen-specific antibody. Antibody levels were not assessed over time for the vaccinated subgroup due to the recent timing of vaccinations.

### Comparison of serum and saliva IgG antibody prevalence and levels

**Figure 3:**
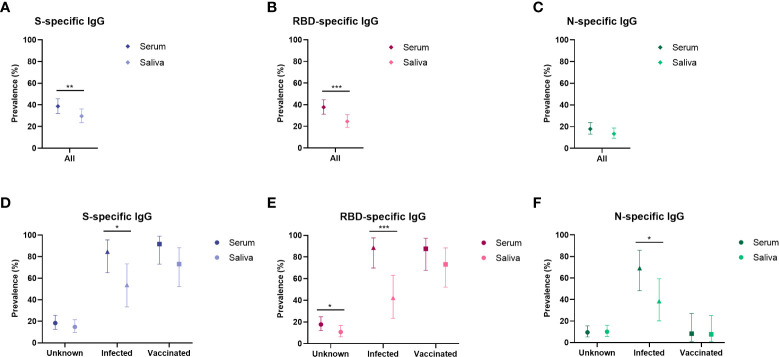
Prevalence of SARS-CoV-2 specific antibodies in serum and saliva compared for population subgroups. Prevalence estimates of antibodies in serum and saliva compared in the total cohort (serum n = 212, saliva n = 216) specific for **(A)** Spike, **(B)** RBD and **(C)** Nucleocapsid, and prevalence shown separately for the unknown group (serum n = 147, saliva n = 149), the infected group (serum n = 24, saliva n = 26) and the vaccinated group (serum n = 26, saliva n = 26) specific for **(D)** Spike, **(E)** RBD and **(F)** Nucleocapsid. Prevalence estimates are the calculated proportion with a value above the determined cut-off. Estimates are shown with 95% confidence intervals. McNemar test was used for differences between paired proportions. S, spike; RBD, receptor binding domain of the spike; N, nucleocapsid; * = p < 0.050, ** = p ≤ 0.010, *** = p ≤0.001.

**Figure 4:**
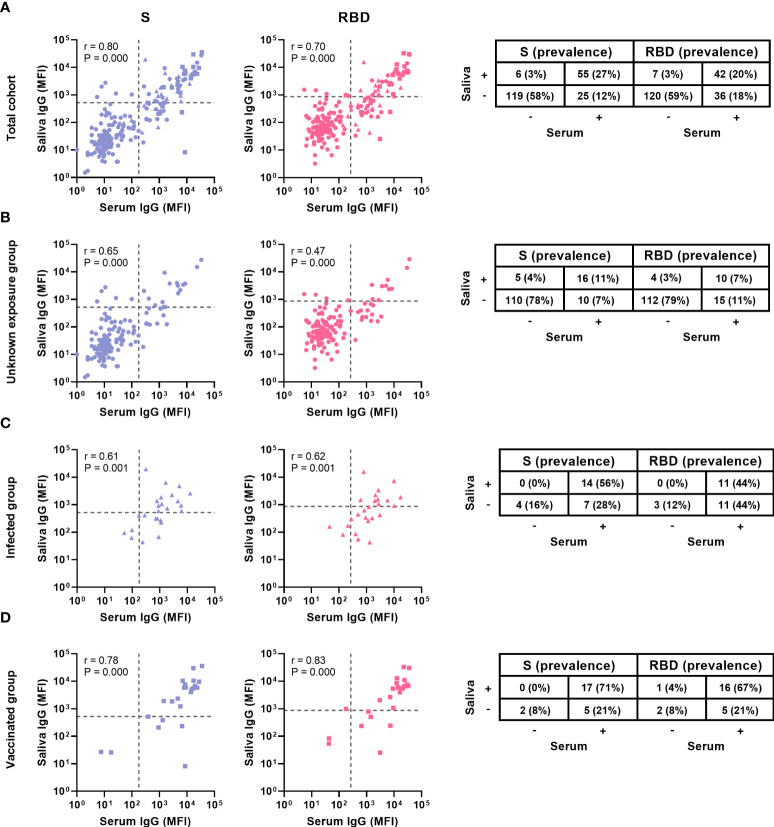
Comparison of serum and saliva antibody levels and prevalence. Levels and prevalence of S- and RBD-specific antibodies of children with paired samples of **(A)** the total cohort (n = 194) **(B)** the unknown exposed group (n = 141), **(C)** the infected group (n = 25) and **(D)** the vaccinated group (n = 24) in serum and saliva (shown on the x and y axis, prevalence is indicated by the percentages). The grey dashed line represents the cut-off for each assay. Spearman’s rank correlations were performed and the coefficient (r) and the P-value are shown for each graph. S, Spike; RBD, receptor binding domain of the spike; MFI, median fluorescence intensity.

We compared the IgG prevalence in all paired serum and saliva samples and detected a significantly lower prevalence in saliva for S- and RBD-specific IgG (P ≤ 0.003), while this was not significantly different for N-specific IgG (P = 0.082, **Figure 3**). When evaluating the concordance between serum and saliva for all three antigens, 20-27% (42-55/205) of children was positive for both serum and saliva SARS-CoV-2 specific IgG, while 12-18% of children was only positive in serum. SARS-CoV-2-specific IgG in saliva could be detected in 54-69% of seropositive children (**Figure 4**). Only 3% (6-6/223) of the cohort was positive in saliva while negative in serum. Further describing this group, saliva SARS-CoV-2 specific IgG was detected in 11% (15/134) of Wantai serum assay negative children (7% of total cohort) and 7% (9/131) of Luminex serum assay negative children (4% of the total cohort). Most of these children reported clinical clues for exposure to SARS-CoV-2 in the form of multiple positive saliva assays, history of vaccination or PCR positive infection, PCR positive infected household members or a combination (5%, 12/223 of the total cohort).

To evaluate the correlation between serum and saliva antibodies, we calculated Spearman’s rank-order correlations. We observed a strong positive correlation between serum and saliva for S- and RBD- specific IgG (r 0.80, P < 0.001 and r 0.70, P < 0.001 respectively, **Figure 4**). For N-specific IgG, which is only elicited by infection and not by vaccination, we found a moderate positive correlation between serum and saliva (r 0.49, P < 0.001, **Supplementary Figure 4**). Saliva IgA only correlated weakly with serum IgG for S-specific antibodies (r 0.3, P < 0.001, **Supplementary Figure 5**)

### Comparison of serum and saliva IgG antibodies in population subgroups

For subgroup analyses, the study cohort was divided into three groups: the infected group (n= 27), only consisting of children with a history of PCR- or rapid antigen test confirmed SARS-CoV-2 infection at least 14 days prior to study inclusion; the vaccinated group (n= 26), only consisting of all children that received at least one vaccination dose at least 14 days prior to study inclusion; and the unknown exposure group (n= 156), consisting of unvaccinated children with no known history of SARS-CoV-2 infection. Six children were both vaccinated and reported a history of SARS-CoV-2 infection and were excluded from subgroup analyses.

We investigated the serum and saliva antibody prevalence separately in the subgroups (**Figure 3**). In the unknown exposure group there was only a significant difference between serum and saliva RBD-specific IgG prevalence (18% and 11% respectively, P = 0.019). In the infected group, there was a significant difference between serum and saliva IgG for all three antigens (P < 0.040). In the vaccinated group there was no significant difference between serum and saliva for all three antigens. The vaccinated group showed the highest concordance between serum and saliva with 67-71% (16-17/24) positive in both compartments (**Figure 4**). In the infected group, 44 – 56% (11-14/25) of children was positive in both serum and saliva. We observed more children with S and RBD-specific serum IgG but no S and RBD-specific saliva IgG in the infected group compared to the vaccinated group (28 – 44% versus 21%, respectively). In all subgroups, only a small proportion (0-4%) was only positive in saliva while negative in serum. Spearman’s rank-order correlations between serum and saliva S-specific IgG were stronger in the vaccinated group compared to the infected group (r = 0.78 versus r = 0.61, respectively, **Figure 4**).

### Associations with demographic and clinical variables

Since prevalence of saliva SARS-CoV-2 IgG was lower compared to serum, we investigated if prevalence was associated with sex, age or comorbidity in the total cohort using a multivariable logistic regression model adjusting also for vaccination and history of infection confirmed by PCR or rapid antigen test. Prevalence of saliva S-specific IgG was higher in girls (40%) compared to boys (19%, P < 0.02). In the multivariable analysis correcting for age, comorbidity, vaccination and infection, sex was a significant predictor for S-specific IgG prevalence in saliva (aOR 2.63, 95% CI 1.24 – 5.58) but not for RBD-specific IgG in saliva nor for S- and RBD-specific IgG in serum (**Table 2**). There was an age-related association with saliva and serum SARS-CoV-2 specific IgG which disappeared after correcting for sex, comorbidity, vaccination and infection. Regarding comorbidity, lower odds for RBD-specific IgG positivity in saliva and S- and RBD-specific IgG in serum were seen for immunocompromised compared to healthy children (aOR 0.23 – 0.25, P < 0.050, **Table 2**). When evaluating associations of variables with SARS-CoV-2 specific IgG in linear regression, sex, age and comorbidity were not associated (data not shown).

**Table 2:** Logistic regressions for serum and saliva IgG prevalence.

Saliva S-specific IgG	Univariable			Multivariable		
	**OR**	**95% CI**	**P**	**aOR**	**95% CI**	**P**
**Female sex**	2.82	1.53 – 5.20	**<.001**	2.63	1.24 – 5.58	**0.012**
**Age**	1.10	1.02 – 1.18	**0.013**	1.05	0.95 – 1.16	0.350
**No comorbidity** **Immunocompromised** **Other illness**	Ref 1.06 1.49	0.44 – 2.61 0.56 – 3.94	0.891 0.426	Ref 0.27 0.80	0.08 – 1.01 0.24 – 2.64	0.051 0.718
**No COVID-19 vaccination** **One dose received** **Two doses received**	Ref 5.31 32.21	1.60 – 17.65 7.13 – 145.54	**0.007** **<.001**	Ref 5.01 43.74	1.26 – 19.93 8.83– 216.76	**0.022** **<.001**
**Previous SARS-CoV-2 infection**	4.48	2.05 – 9.79	**<.001**	5.87	2.40 – 14.39	**<.001**
						
**Saliva RBD-specific IgG**	**Univariable**			**Multivariable**		
	**OR**	**95% CI**	**P**	**aOR**	**95% CI**	**P**
**Female sex**	2.21	1.16 – 4.19	**0.015**	2.10	0.96 – 4.61	0.064
**Age**	1.07	0.99 – 1.15	0.086	1.03	0.92 – 1.14	0.630
**No comorbidity** **Immunocompromised** **Other illness**	Ref 0.87 1.64	0.34 – 2.24 0.60 – 4.51	0.773 0.335	Ref 0.25 1.00	0.06 – 0.99 0.30 – 3.36	**0.048** 0.998
**No COVID-19 vaccination** **One dose received** **Two doses received**	Ref 5.07 27.02	1.53 – 16.78 7.41 – 98.54	**0.008** **<.001**	Ref 5.70 41.91	1.44 – 22.64 10.06 – 174.68	**0.013** **<.001**
**Previous SARS-CoV-2 infection**	2.87	1.31 – 6.28	**0.008**	4.59	1.82 – 11.57	**0.001**
						
**Serum S-specific IgG**	**Univariable**			**Multivariable**		
	**OR**	**95% CI**	**P**	**aOR**	**95% CI**	**P**
**Female sex**	2.17	1.24 – 3.83	**0.007**	1.82	0.86 – 3.83	0.116
**Age**	1.10	1.03 – 1.17	**0.007**	1.08	0.98 – 1.19	0.133
**No comorbidity** **Immunocompromised** **Other illness**	Ref 1.02 1.62	0.45 – 2.32 0.65 – 4.04	0.962 0.298	Ref 0.24 1.03	0.07 – 0.80 0.34 – 3.10	**0.020** 0.957
**No COVID-19 vaccination** **One dose received** **Two doses received**	Ref 25.67 37.33	3.23 – 203.77 4.83 – 288.64	**0.002** **<.001**	Ref 32.26 58.52	3.56 – 292.73 7.05 – 485.74	**0.002** **<.001**
**Previous SARS-CoV-2 infection**	16.20	5.42 – 48.45	**<.001**	22.96	6.98 – 75.54	**<.001**
						
**Serum RBD-specific IgG**	**Univariable**			**Multivariable**		
	**OR**	**95% CI**	**P**	**aOR**	**95% CI**	**P**
**Female sex**	2.02	1.15 – 3.55	**0.015**	1.56	0.73 – 3.32	0.249
**Age**	1.09	1.02 – 1.17	**0.009**	1.07	0.97 – 1.19	0.169
**No comorbidity** **Immunocompromised** **Other illness**	Ref 0.99 1.51	0.43 – 2.24 0.60 – 3.75	0.973 0.380	Ref 0.23 0.93	0.07 – 0.79 0.30 – 2.82	**0.019** 0.893
**No COVID-19 vaccination** **One dose received** **Two doses received**	Ref 11.98 38.34	2.54 – 56.54 4.96 – 296.50	**0.002** **<.001**	Ref 14.44 64.98	2.54 – 81.96 7.81 – 540.76	**0.003** **<.001**
**Previous SARS-CoV-2 infection**	24.26	7.08 – 83.19	**<.001**	35.95	9.63 – 134.22	**<.001**

Uni- and multivariable regression values with serum and saliva IgG levels above the cut-off for positivity as the dependent variable. Odds ratios (OR) and adjusted odds ratios (aOR) with 95% confidence intervals (CI) for variables included in the regression models are reported. Variables reaching statistical significance are presented in bold (P < 0.050). Study inclusion at least 14 days after PCR- or rapid antigen test confirmed SARS-CoV-2 infection or first dose of COVID-19 vaccination was considered sufficient for inclusion in the regression models.

## Discussion

We detected SARS-CoV-2 specific IgG and IgA in both serum and saliva in a substantial group of children attending regular medical services. There was heterogeneity in the humoral response with an overall higher prevalence in serum compared to saliva. Vaccinated children showed higher correspondence between serum and saliva positivity than previously infected children. Moreover, girls had higher odds for saliva SARS-CoV-2 specific IgG compared to boys and immunocompromised children had lower odds for both serum and saliva IgG. Understanding the differences between systemic and mucosal humoral responses provides insight for the application of mucosal antibody assays.

Similar to our previous study, we observed that determining the prevalence for SARS-CoV-2 specific mucosal IgA was less accurate than for IgG, and that salivary IgA correlates poorly to serum IgA. This is consistent with other studies emphasizing the lower sensitivity for saliva IgA to detect PCR-positive patients, more nonspecific and cross-reactive binding of saliva IgA and a shorter durability as compared to saliva IgG (2, 13–15, 29). The IgG antibody prevalence among Dutch children attending regular medical services has increased sharply from 3 – 4% in serum and saliva in our previous 2020 study to 30 – 38% in 2021 (22). In the corresponding period of 2021, seroprevalence among Dutch adult blood donors in a national survey increased from 31% to 95% due to vaccination programs and increasing infection rates. Compared to adults, children experience more frequent asymptomatic infections. With a large proportion of infections remaining asymptomatic, as was also seen in our cohort of children, antibody assays are an important addition to symptom driven PCR testing to assess infection rates and estimate immunity in a population.

We report heterogeneity between antibodies targeting different antigens, with a significantly lower prevalence for N-specific antibodies, also when analyzing only unvaccinated children. An important factor in explaining these differences could be the variation in time kinetics. We potentially observed a more rapid decline over time for N-specific IgG compared to S and RBD-specific IgG, measured up to more than one year after infection. Several longitudinal studies corroborate a faster decline of N-specific IgG compared to S- and RBD-specific IgG, showing a significant drop in N-specific IgG several months after infection in serum and saliva (30–32). Importantly, a study of antibody dynamics showed a several‐fold variation between individuals in half‐lives of SARS-CoV-2 specific IgG (33). In addition, N-specific antibodies often seem to be absent in asymptomatic patients (34, 35). Therefore, besides a more rapid decline of specific antibodies over time, there could be heterogeneity between individuals in the elicitation and preservation of specific humoral responses in the first place. In a broader perspective, an analysis of peripheral blood mononuclear cells showed substantial variability between healthy individuals in numbers of naïve B cells, plasmablasts, memory CD4^+^ T cells, effector CD8^+^ T cells and mucosa-associated innate T-cells, suggesting individual tendencies towards a more pronounced B-cell mediated or T-cell mediated response to pathogens (36).

In line with our previous study, heterogeneity is also shown between the mucosal and systemic compartments (22). Although serum and saliva IgG were both detectable in most SARS-CoV-2-specific IgG positive children, saliva IgG prevalence was lower than serum IgG prevalence. Longitudinal studies suggest a difference in time kinetics, with slightly lower percentages of saliva IgG positive individuals remaining positive after 9 to 15 months follow-up compared to serum IgG in mild adult COVID-19 patients (72 – 88% in saliva compared to 89 – 96% in serum) (15, 37). We also report a greater difference between serum and saliva IgG in the infected group as compared to the vaccinated group. Median time since last exposure or vaccination respectively was six-fold longer in the infected group as compared to the vaccinated group which is likely to contribute to this difference. Considering that some studies measure similar durability for serum and mucosal SARS-CoV-2 IgG (38, 39), heterogeneity in the response itself could also explain the differences between compartments. If exposure to the virus does not elicit identical humoral immune responses in all individuals, this may explain the lower saliva prevalence reported in several COVID-19 cohorts (18, 40).

To explore the value of mucosal samples, it is crucial to identify which factors can predict certain systemic or mucosal humoral responses. We showed an association between female sex and saliva SARS-CoV-2-specific antibody positivity in children. This association was not found for serum SARS-CoV-2-specific antibody positivity, suggesting that the mucosal compartment may be more prone to sex-related differences than the systemic compartment. Differences between male and female immunity – although predominantly after sexual maturation – have been described with stronger antibody responses, higher basal Ig titers and higher number of B cells in females (41). Our lack of sexual development data, such as Tanner scores, therefore imposes an important limitation in evaluating associations with sex and this information should be collected in future pediatric antibody studies. In SARS-CoV-2 infection, adult males show a slower, more gradual increase of RBD-specific IgG in the acute phase and a faster decline of S- and RBD-specific and neutralizing antibodies compared to females (32, 42–44). Of note, in our study this association with female sex was only apparent in S-specific IgG but not in RBD- and N-specific IgG. This may be explained by slightly lower prevalence of RBD- and N- compared to S-specific IgG thus lacking sufficient numbers to reach statistical significance. Alternatively, associations of antibodies with sex and comorbidity could indeed be antigen-specific, and thus may only be present for S-specific IgG. Supporting this latter hypothesis, similar findings have been reported for convalescent patients showing significantly higher S1-specific antibody prevalence in females compared to males, whereas the difference between sexes in RBD- and N-specific antibody prevalence was not significant (45). This possibility of antigen-specific associations should be taken into consideration in future studies. Moreover, we observed lower odds for immunocompromised compared to healthy children for saliva RBD-specific IgG and serum S- and RBD-specific IgG, indicating a possible higher risk for more frequent or severe reinfection. As immunocompromised patients can show adequate serum and saliva responses after two vaccinations, the clinical relevance of lower humoral responses is probably largely influenced by the type of disease or immunomodulating drugs, previous SARS-CoV-2 immunity and number and timing of vaccinations (46, 47).

The relevance of non-invasive mucosal antibody assays is increasing in this phase of the pandemic because of the increasing vaccination rates and decreasing hospital admission rates. Described in this study and in several other cohorts, the humoral response after vaccination shows a higher correlation between the mucosal and systemic humoral IgG responses when compared to natural infection (3, 48, 49). This suggests a potential use for saliva antibodies to monitor vaccine response. Additionally, in line with our previous study we found Wantai seronegative children with mucosal antibodies. Considering the 96% specificity, this could be explained by possible false positivity. However, some COVID-19 cohorts have similarly shown patients with mucosal antibodies without seroconversion (40, 50). Furthermore, most of our seronegative children with mucosal antibodies showed convincing clinical clues for SARS-CoV-2 exposure. Saliva assays are less sensitive than serum assays but they can potentially identify seronegative convalescent patients with saliva antibodies.

In children, this is the first study evaluating associations of mucosal antibodies and demographic variables. However, there are several limitations. An important limitation is the lack of complete data regarding exposure to SARS-CoV-2 as we did not perform structured PCR testing in the participants. By means of written and verbal questionnaires, we have questioned all participants whether they were aware of a previous SARS-CoV-2 infection. This study investigated associations of demographic variables with antibody positivity by including known previous PCR positivity and vaccination status in the models. In this way, we aimed to investigate differences in humoral responses instead of differences in exposure to SARS-CoV-2. The inclusion of previous infection and vaccination data in the regression models did indeed change the outcome. Unfortunately, data on COVID-19 severity was too scarce to include in the regression analyses. Finally, our study cohort included as large proportion of immunocompromised children and children using immunomodulating drugs, which allowed investigation of the effect of this variable, but is also a limitation for the general applicability of our results.

In conclusion, this cross-sectional study confirms that humoral immunity can be detected in saliva, preferably with S- and RBD-specific IgG. Sex and immunocompromisation may affect antigen-specific SARS-CoV-2 IgG antibody prevalence in children. Differences between infection and vaccination, between sexes and between immunocompromised and healthy children should be further investigated and considered when choosing systemic or mucosal antibody measurement. Future studies may also focus on longitudinal analysis of antibody levels in repeated saliva samples from children and the protective capacity of saliva antibodies. On a population level, saliva-based assays can be useful for identifying vulnerable SARS-CoV-2 naive populations and vaccine responses in a non-invasive manner.

## Data availability statement

The raw data supporting the conclusions of this article will be made available by the authors upon request, without undue reservation.

## Ethics statement

The studies involving human participants were reviewed and approved by Medical ethics committee of the Amsterdam University Medical Centers. Written informed consent to participate in this study was provided by the participants’ legal guardian/next of kin.

## Author contributions

Study design: MK, DP, MB. Inclusion and data collection: MK, BA, FG, MF, FP, MR, HE. Conducting experiments: MG, KT, JR, MS, TR. Data analysis: MK, MG. Writing the manuscript: MK, MG. Reviewing and editing the manuscript: all authors. First author order was determined on the basis of contributions to study conception and design. All authors contributed to the article and approved the submitted version.

## Funding

The Contribute Foundation (226) and the Tergooi Scientific Committee (21.03).

## Acknowledgments

This study would not have been possible without the financial support of the Contribute Foundation and the Tergooi Scientific committee. We are thankful for the hard work and enthusiasm of the medical students and physicians supporting patient inclusion and laboratory staff for sample preparation. Specifically, we thank Mariette Ravenhorst and the research team of the RECoVERD/VIS- cohort study for providing control samples and Eli Vlessing for his creative efforts. Finally, we are most grateful to all the parents and children participating in this study.

## Conflict of interest

The authors declare that the research was conducted in the absence of any commercial or financial relationships that could be construed as a potential conflict of interest.

## Publisher’s note

All claims expressed in this article are solely those of the authors and do not necessarily represent those of their affiliated organizations, or those of the publisher, the editors and the reviewers. Any product that may be evaluated in this article, or claim that may be made by its manufacturer, is not guaranteed or endorsed by the publisher.
