# Supplementary material for: Differences in systemic and mucosal SARS-CoV-2 antibody prevalence in a prospective cohort of Dutch children
Source: Front Immunol. 2022 Sep 9;13:976382. doi: 10.3389/fimmu.2022.976382 (PMC9500453; doi:10.3389/fimmu.2022.976382)
Supplement: Supplementary file 1 [file DataSheet_1.docx]

# Supplementary materials

|  | Sensitivity | Sensitivity 95%CI | Specificity | Specificity 95%CI |
| --- | --- | --- | --- | --- |
| Serum, S, IgG | 97% | 92.48% to 99.28% | 96% | 93.60% to 98.06% |
| Serum, RBD, IgG | 96% | 91.25% to 98.61% | 91% | 87.65% to 94.21% |
| Serum, N, IgG | 99% | 95.16% to 99.95% | 94% | 90.14% to 95.92% |
| Saliva, S, IgG | 77% | 66.05% to 85.41% | 88% | 76.20% to 94.38% |
| Saliva, RBD, IgG | 73% | 61.46% to 81.88% | 96% | 86.54% to 99.29% |
| Saliva, N, IgG | 80% | 69.18% to 87.70% | 88% | 76.20% to 94.38% |
| Saliva, S, IgA | 71% | 59,43% to 80,38% | 88% | 76,20% to 94,38% |
| Saliva, RBD, IgA | 75% | 64,04% to 84,01% | 82% | 69,20% to 90,23% |
| Saliva, N, IgA | 45% | 33,77% to 56,62% | 80% | 66,96% to 88,76% |

**Supplementary table 1. Specificity and sensitivity of Luminex assays to detect previous PCR positive SARS-CoV-2 infection**

**
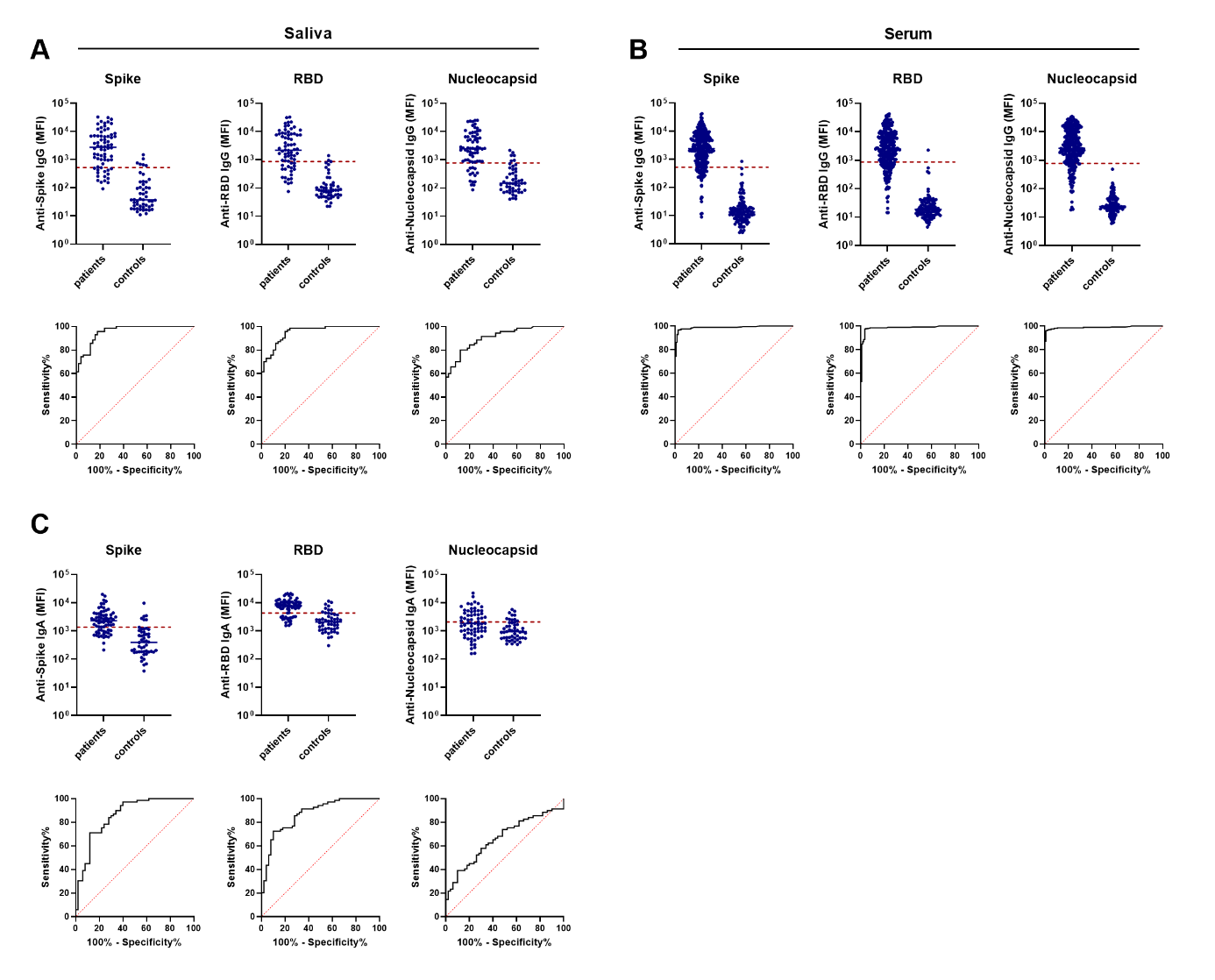
**

**Supplementary Figure 1. ROC analysis for Luminex assays**(A) ROC analysis corroborating our previously determined cut-off (reference 22) for the Luminex assay for IgG in saliva with pre-pandemic saliva of children (n = 50) and SARS-CoV-2 infected adults (n = 70). (B) ROC analysis corroborating our previously determined cut-off (reference 22) for the Luminex assay for IgG in serum with serum of pre-pandemic (n = 113) and PCR-confirmed SARS-CoV-2 infected (n = 282) adults. The red dotted lines are the cut-offs. (C) ROC analysis to determine a new cut-off for the Luminex assay for IgA in saliva using pre-pandemic saliva of children (n = 50) and SARS-CoV-2 infected adults (n = 70). Cut-offs were selected as the highest sensitivity achievable with a specificity of at least 80% and indicated as red dotted lines. S = spike, RBD = receptor binding domain of the spike, N = nucleocapsid, MFI = median fluorescence intensity.


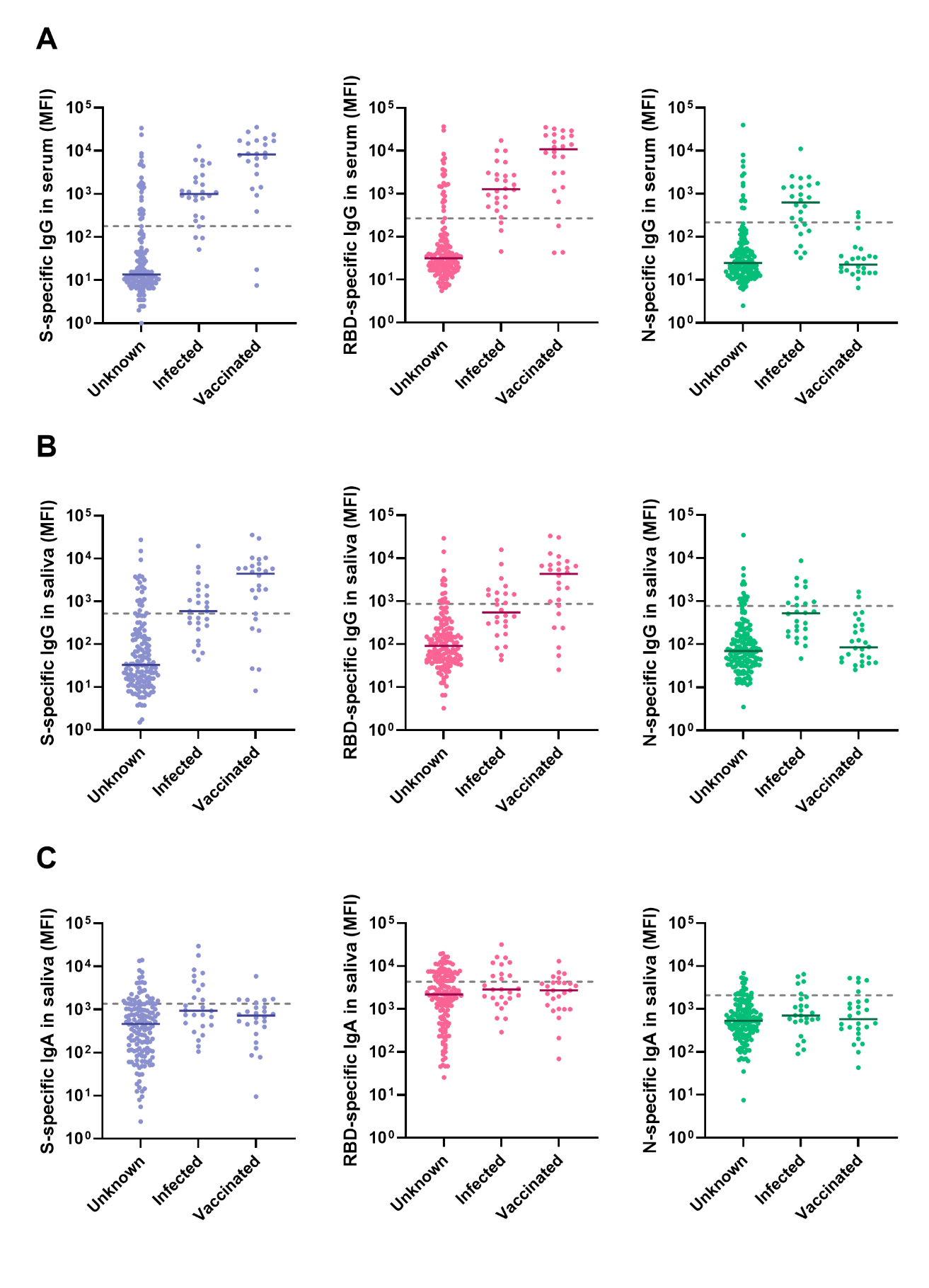
**Supplementary Figure 2. Levels of SARS-CoV-2 specific IgG and IgA in serum and saliva**(A) Levels of S, RBD and N-specific IgG in serum, presented separately for the unknown exposure group (n = 147), the infected group (n = 26) and the vaccinated group (n = 24). (B) Levels of S-, RBD- and N-specific IgG in saliva, presented separately for the unknown exposure group (n = 149), the infected group (n = 26) and the vaccinated group (n = 26). (C) Levels of S-, RBD- and N-specific IgA in saliva, presented separately for the unknown exposure group (n = 149), the infected group (n = 26) and the vaccinated group (n = 26). The grey dotted lines are the cut-offs for positivity. S = spike, RBD = receptor binding domain of the spike, N = nucleocapsid, MFI = median fluorescence intensity.

**
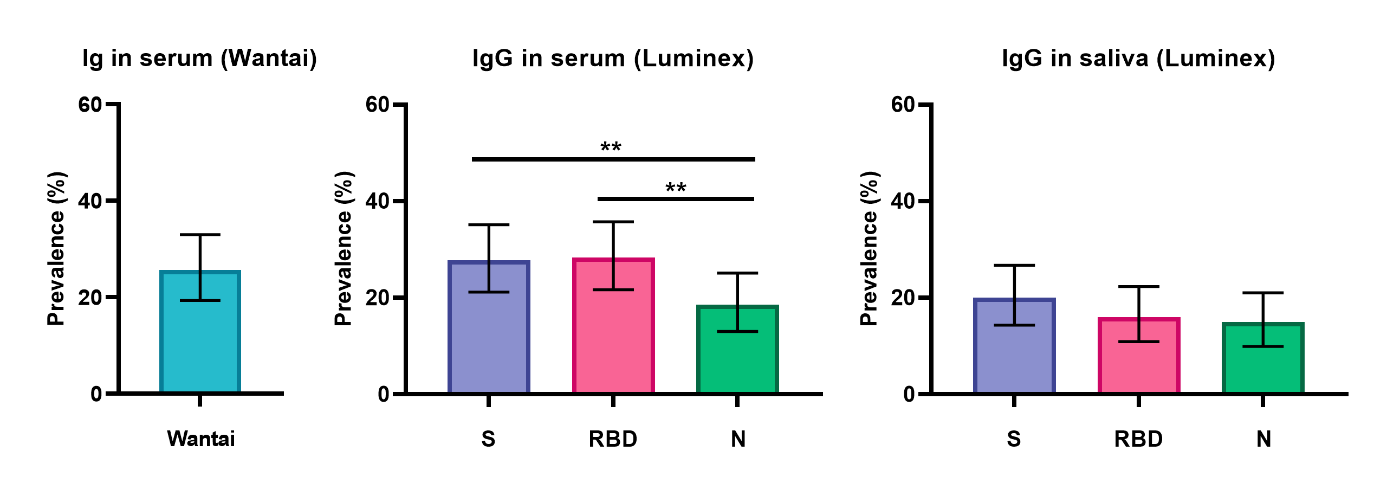
**

**Supplementary Figure 3. Prevalence of SARS-CoV-2 specific antibodies in serum and saliva in unvaccinated children**

Prevalence estimates of RBD-specific antibodies in serum using the Wantai assay (n = 171) and of S-, RBD- and N-specific antibodies using the Luminex assay in serum (n = 173) and saliva (n = 175) only for unvaccinated children (in total n = 182). Prevalence estimates are the calculated proportion with a value above the determined cut-off out. Estimates are shown with 95% confidence intervals. McNemar test was used for differences between paired proportions. S = spike, RBD = receptor binding domain of the spike, N = nucleocapsid, ** = P < 0.01.

**
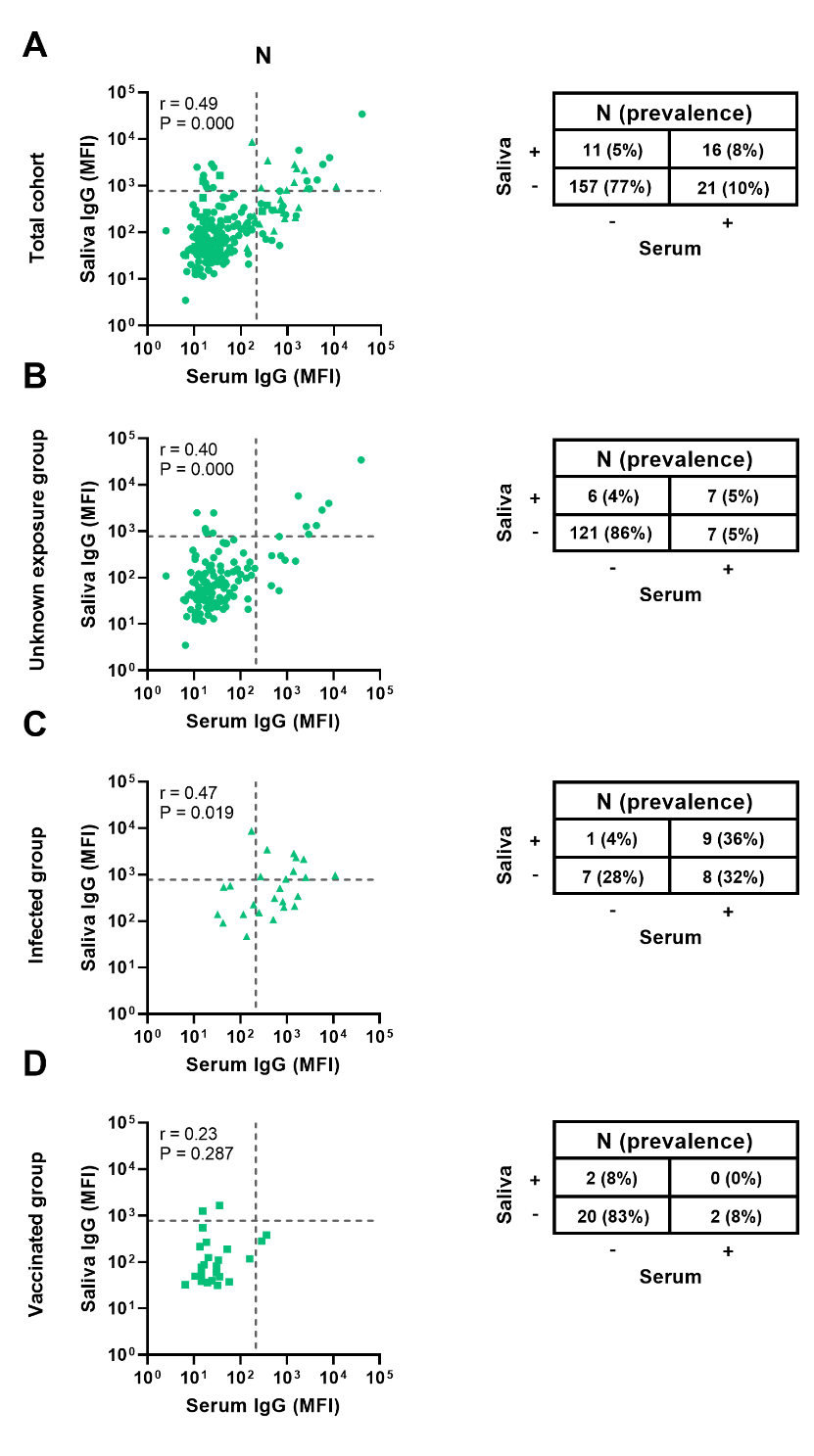
**

**Supplementary Figure 4. Comparison of N-specific serum and saliva antibody levels and prevalence**Levels and prevalence of N-specific antibodies of children with paired samples of (A) the total cohort (n = 194) (B) the unknown exposed group (n = 141), (C) the infected group (n = 25) and (D) the vaccinated group (n = 24) in serum and saliva (shown on the x and y axis, prevalence is indicated by the percentages). The grey dashed line represents the cut-off for each assay. Spearman’s rank correlations were performed and the coefficient (r) and the P-value are shown for each graph. N = nucleocapsid, MFI = median fluorescence intensity.


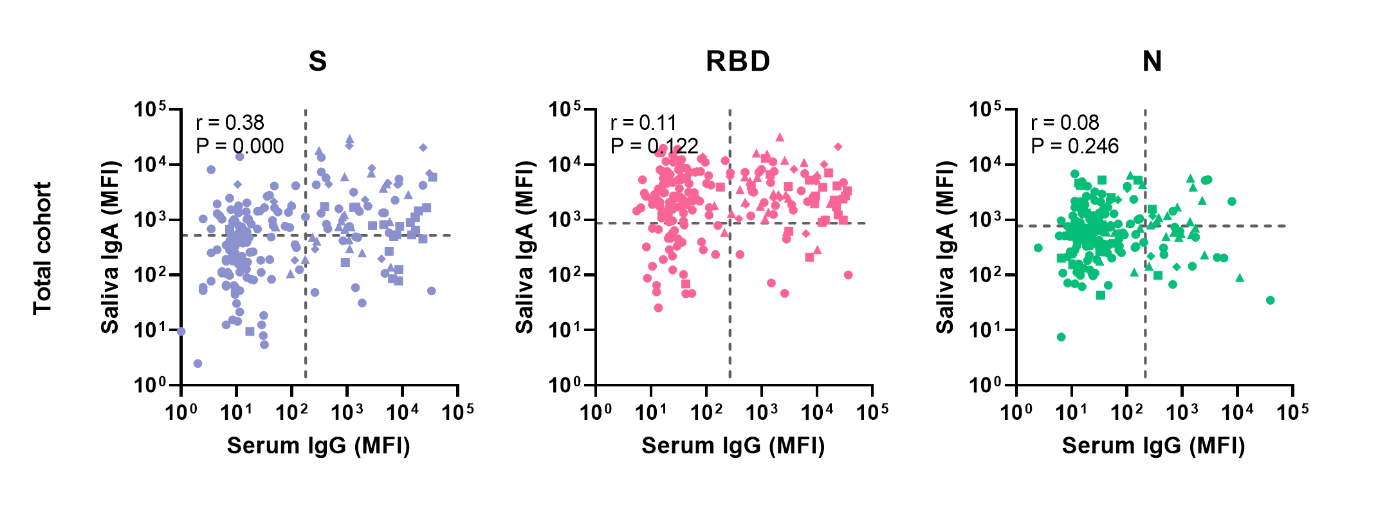


**Supplementary Figure 5. Comparison of saliva IgA and serum IgG levels and prevalence**Levels and prevalence of S, RBD and N-specific antibodies of children with paired samples of the total cohort (n = 194) in serum and saliva (shown on the x and y axis, prevalence is indicated by the percentages). The grey dashed line represents the cut-off for each assay. Spearman’s rank correlations were performed and the coefficient (r) and the P-value are shown for each graph. S = spike, RBD = receptor binding domain of the spike, N = nucleocapsid, MFI = median fluorescence intensity.
